# Transcriptome-based identification of key actin-binding proteins associated with high metastatic potential in breast cancer

**DOI:** 10.3389/fmolb.2024.1440276

**Published:** 2024-08-30

**Authors:** Christian Müller, Leticia Oliveira-Ferrer, Volkmar Müller, Barbara Schmalfeldt, Sabine Windhorst

**Affiliations:** ^1^ Bioinformatics Core, University Medical Center Hamburg-Eppendorf, Hamburg, Germany; ^2^ Department of Gynecology, University Medical Center Hamburg-Eppendorf, Hamburg, Germany; ^3^ Department of Biochemistry and Signal Transduction, University Medical Center Hamburg-Eppendorf, Hamburg, Germany

**Keywords:** actin, metastasis, actin-binding proteins, bioinformatics, breast cancer

## Abstract

**Introduction:**

Actin-binding proteins (ABPs) are essential for the regulation of morphological plasticity required for tumor cells to metastasize. The aim of this study was to perform an unbiased bioinformatic approach to identify the key ABPs significantly associated with the metastatic potential of breast cancer cells.

**Methods:**

Microarray data from 181 primary breast cancer samples from our hospital were used, and all genes belonging to the Gene Ontology term actin cytoskeleton organization were obtained from QuickGO. Association with metastasis-free survival probability was tested using Cox proportional hazards regression, and pairwise co-expression was tested by Pearson correlations. Differential expression between different subgroups was analyzed using Wilcoxon tests for dichotomous traits and Kruskal–Wallis tests for categorical traits. Validation was performed using four publicly available breast cancer datasets.

**Results:**

*ARHGAP25* was significantly associated with a low metastatic potential, and *CFL1*, *TMSB15A*, and *ACTL8* were significantly associated with a high metastatic potential. A significantly higher expression of *CFL1*, *TMSB15A,* and *ACTL8* mRNA was found in the more aggressive Her2-positive and triple-negative subtypes as well as in ER-negative samples. Also, these genes were co-expressed in the same tumors. However, only mRNA levels of *CFL1* were increased in pN1 compared to pN0 patients. External validation revealed that *CFL1* and *TMSB15A* had significant associations with consistent hazard ratios in two breast cancer cohorts, and among these, *CFL1* exhibited the highest hazard ratios.

**Conclusion:**

*CFL1* showed the strongest correlation with the metastatic potential of breast tumors. Thus, targeted inhibition of *CFL1* might be a promising approach to treat malignant breast cancer cells.

## Introduction

Distant metastasis is the leading cause of cancer-related death in breast cancer and most other solid tumors, and currently, no therapy is available to cure patients suffering from metastasis (Fares et al., 2020). Therefore, the identification and inhibition of metastasis-promoting genes in cancer populations is of great clinical relevance. In order to form distant metastases, tumor cells must undergo strong morphological changes to escape from the primary tumor, invade the tissue and blood vessels, and finally, extravasate from the vessels and form new metastases. This morphological plasticity requires remodeling of the actin cytoskeleton, which is controlled by many actin-binding proteins (ABPs). During amoeboid and mesenchymal migration, as well as during invasion, the tumor cells form different kinds of protrusions at the leading edges, requiring the formation, bundling, and cross-linking of actin filaments (F-actin) at the tips of the cells. Also, F-actin is cleaved to provide new actin monomers (G-actin) for F-actin elongation, and actin-myosin contraction provides the force needed for cell migration and invasion. In addition, some ABPs only bind to G-actin; they sequester monomeric actin or promote its polymerization to F-actin (Olson and Sahai, 2009; Pollard, 2016; Lappalainen et al., 2022).

The ABPs regulating these F-actin dynamics include proteins promoting elongation or formation of F-actin (Arp2/3-complex, formins, vasodilator-stimulated phosphoprotein (VASP)), bundling and/or cross-linking F-actin (Fascin, Plastins, Actinins, Inositol 1,4,5-trisphosphate 3-kinase-A (ITPKA)), capping actin (CAP-Z, gelsolin), binding to actin monomers (Thymosin, Profilin), or severe F-actin (Cofilin and Gelsolin). Most of these ABPs are inactive in resting cells and are stimulated by the RhoGTPases Rac-1, Cdc42, and Rho-A, but some of them are constitutively targeted to F-actin (Pollard, 2016; Windhorst et al., 2017).

Because of the important role of actin dynamic regulation for cancer cell metastasis, the expression and/or activity of several ABPs are upregulated in malignant cancer cells, and it could be shown that this upregulation is associated with cancer cell metastasis. These proteins include the actin-bundling proteins Fascin-1, L-Plastin, Cortactin, and ITPKA, the ENA/VASP proteins, the F-actin severing protein Cofilin 1, and many more (Olson and Sahai, 2009; Stevenson et al., 2012; Windhorst et al., 2017; Izdebska et al., 2020). Interestingly, most of these proteins are not expressed in the corresponding normal tissue and are thus suitable targets for tumor therapy (Stevenson et al., 2012; Windhorst et al., 2017; Izdebska et al., 2020).

An unbiased bioinformatic approach was conducted in this study to reveal which ABPs are most frequently expressed in breast cancer cells with high metastatic potential. Our goal was to identify those ABPs whose upregulation correlates most strongly with the potential of breast cancer cells to form metastases. Thereby, we intended to identify the most effective ABP targets for anti-cancer therapy.

## Methods and methods

### Discovery cohort

The discovery (in-house) cohort included microarray data from 194 primary, untreated breast cancer patients, of whom metastasis-free survival data were available for 181 (Milde-Langosch et al., 2014). Briefly, all patients were treated at the University Medical Centre Hamburg-Eppendorf, Germany, Department of Gynecology between 1991 and 2002 and gave written approval for the utilization of their tissue samples and the reviewing of their medical records according to our investigational review board and ethics committee guidelines (Ethik-Kommission der Ärztekammer Hamburg, #OB/V/03). RNA was isolated from snap-frozen tissue samples, quantified using an Affymetrix (Santa Clara, CA, United States) HG-U133A array on a GeneChip System, and pre-processed as described elsewhere (Milde-Langosch et al., 2014).

### Validation cohorts

Suitable validation cohorts with transcriptome data from primary breast cancer samples with follow-up of metastasis-free survival over at least 5 years were retrieved from the Gene Expression Omnibus (GEO) (Barrett et al., 2013). Four cohorts were identified and used for validation of the discovery findings, including GSE11121 (Schmidt et al., 2008), GSE6532 (Loi et al., 2007), GSE 2034 (Wang et al., 2005), and GSE21653 (Sabatier et al., 2011).

### Pre-processing of microarray data

The pre-processing and data analysis were performed in *R* version 4.2.3. Expression intensities of the discovery cohort were log_2_-transformed and quantile-normalized. Microarray data from the validation cohorts were retrieved from GEO using the *R/Bioconductor* package *GEOquery* (Davis and Meltzer, 2007). The optimal pre-processing strategy for each dataset was evaluated for the effect on global variation based on principal component analysis (PCA). Probe intensities were log2-transformed and quantile-normalized for the studies GSE11121, GSE2034, and GSE21653. In the GSE6532 dataset, the PCA showed strong clusters of different batches represented by the variable *characteristics_ch1.2,* which was subsequently used as a batch variable in the batch effect removal function *ComBat* (Johnson et al., 2007) from the *R/Bioconductor* package *sva* (Leek et al., 2012).

### Survival analysis and candidate gene identification

All genes belonging to the Gene Ontology term *“actin cytoskeleton organization”* (GO:0030036) were obtained from QuickGO (Binns et al., 2009) and filtered for presence in the transcriptome dataset of the discovery cohort. The remaining list was manually filtered for those directly interacting with actin or belonging to the RhoGTPases and their regulators. The mRNA of each ABP was tested for association with metastasis-free survival probability using Cox proportional hazards regression adjusted for age at baseline examination. Genes with a false discovery rate (FDR) ≤ 0.05 were considered significant and considered potential candidates. The mRNA of these genes was tested for association with metastasis-free survival probability in the validation cohorts. Age at baseline examination was only used as a covariate in the Cox model in GSE6532 and GSE21653 because it was not available in GSE11121 and GSE 2034. Genes with a *p*-value ≤0.05 and a consistent effect direction, compared to the discovery, were considered candidates. Candidate gene expression was stratified based on the first quartile for hazard ratios smaller than one, and otherwise, the third quartile was used and visualized as Kaplan–Meier curves, including *p*-values from the log-rank tests.

### Differential gene expression and co-expression analysis

The mRNA of each potential candidate was tested for differential expression between different subgroups of the discovery cohort. Wilcoxon tests were applied for dichotomous traits, and Kruskal–Wallis tests were applied for categorical traits. Pairwise co-expression of all candidate genes was tested using Pearson correlations.

## Results

### Study design and identification of APBs significantly correlated with the metastatic potential of breast cancer cells

**FIGURE 1:**
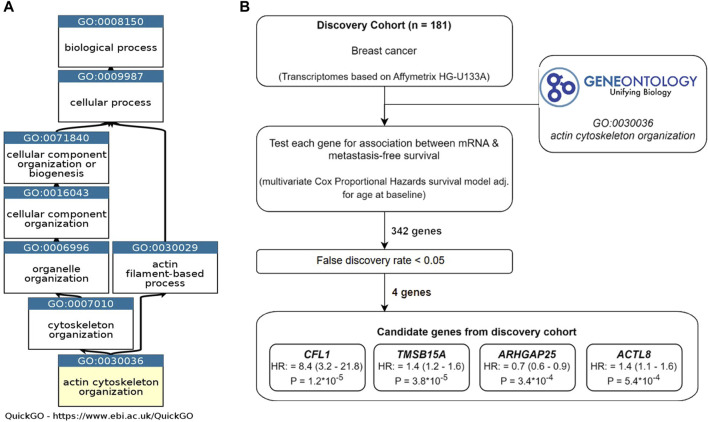
Study design of the discovery phase. **(A)** Ancestor chart of the Gene Ontology term “Actin cytoskeleton organization” (GO:0030036), which was used to select genes for the analysis. **(B)** Workflow and results for the discovery phase.

In order to identify the set of APBs upregulated in highly metastatic breast cancer cells, all annotations for the Gene Ontology term “actin cytoskeleton organization” (GO:0030036) were obtained from *QuickGO* (Binns et al., 2009) (Figure 1A). This category contained 666 unique genes, which were filtered for the presence in our transcriptome dataset derived from 181 breast cancer patients. Thereafter, a list of 342 genes was manually curated by selecting those directly interacting with actin (ABPs) or belonging to the RhoGTPases and their regulators (Figure 1B).

The in-house transcriptome dataset was used as a discovery cohort, and all 342 ABP-related genes were screened for associations to metastasis-free survival using Cox proportional hazards regression adjusted for age at baseline examination. For evaluation, we defined a false discovery rate (FDR) ≤ 0.05. Interestingly, only four genes were considered significant: Cofilin 1 (*CFL1)*, Thymosin beta15A (*TMSB15A*), Rho GTPase activating protein 25 (*ARHGAP25*), and actin like 8 (*ACTL8*). Among these, a high mRNA expression of *CFL1, TMSB15A, and ACTL8* was positively associated with metastasis formation, whereas *ARHGAP25* was associated with lower metastatic rates.

**FIGURE 2:**
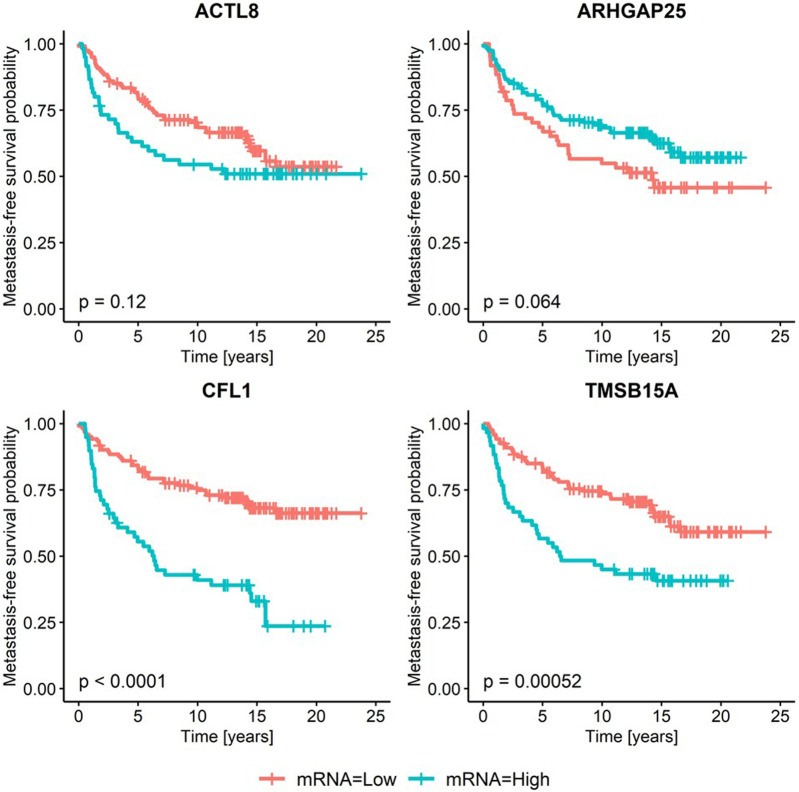
Expression of candidate ABPs in relation to metastasis-free survival. The mRNA expression of candidate genes was stratified based on the second tertile for genes with a hazard ratio >1 (*ACTL8*, *CFL1*, *TMSB15A*) and based on the first tertile if the hazard ratio was <1 (*ARHGAP25*) and the relationship to metastasis-free survival probability was depicted in Kaplan–Meier curves. *p*-values refer to the log-rank test after dichotomization.

Further, patients were divided into quartiles according to the mRNA level of each candidate gene, and Kaplan–Meier analyses and log-rank tests were performed using cut-offs described in materials and methods. Here, a significant association with metastasis-free survival was confirmed for *CFL1 and TMSB15A,* while no prognostic value could be observed for *ACTL8* and *ARHGAP25* (Figure 2).

In summary, among 342 genes coding for ABPs, four are significantly associated with the probability of breast tumors forming distant metastases.

### Co-expression of ABPs and impact on metastasis formation

**FIGURE 3:**
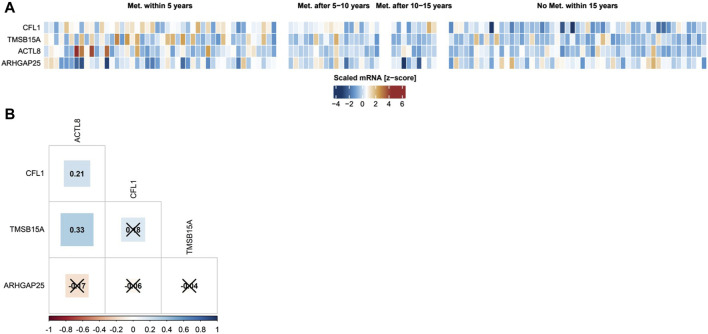
Cluster and co-expression analysis of ABPs. **(A)** The four ABPs whose mRNA expression significantly correlates with the metastatic potential of breast cancer cells were clustered according to the time-course of metastasis formation. **(B)** Pairwise correlations between mRNA expression of ABPs in tumor samples of the discovery cohort. Pearson correlation coefficients with a Bonferroni-corrected *p*-value >0.05 are crossed out.

To analyze the relevance of ABP co-expression for malignancy in breast cancer, cluster analysis including all four candidates was performed within the following four subcohorts: Patients who developed metastasis before 5, between 5–10, between 10–15, or after 15 years. In Figure 3A each rectangle represents one patient, and dark brown represents a high ABP mRNA level, while dark blue represents a low ABP mRNA level. The result of this analysis revealed a clustering of high *CFL1*, T*MSB15A, and ACTL8* in patients who developed metastasis within 5 years, and low levels in patients who did not suffer from metastasis within 15 years. For *ARHGAP25,* we observed the opposite pattern.

In addition, correlation analysis of ABP transcripts revealed a significant positive association between *ACTL8* with *CFL1* and *TMSB15A,* suggesting the co-expression of these proteins in highly metastatic tumors. On the other hand, *CFL1* and *TMSB15A* were not co-expressed, and *ARHGAP25* did not show any co-expression with the other ABPs. Thus, mRNAs of ABPs show distinct co-expression profiles (Figure 3B).

### Correlation of ABPs with breast cancer subtypes

**FIGURE 4:**
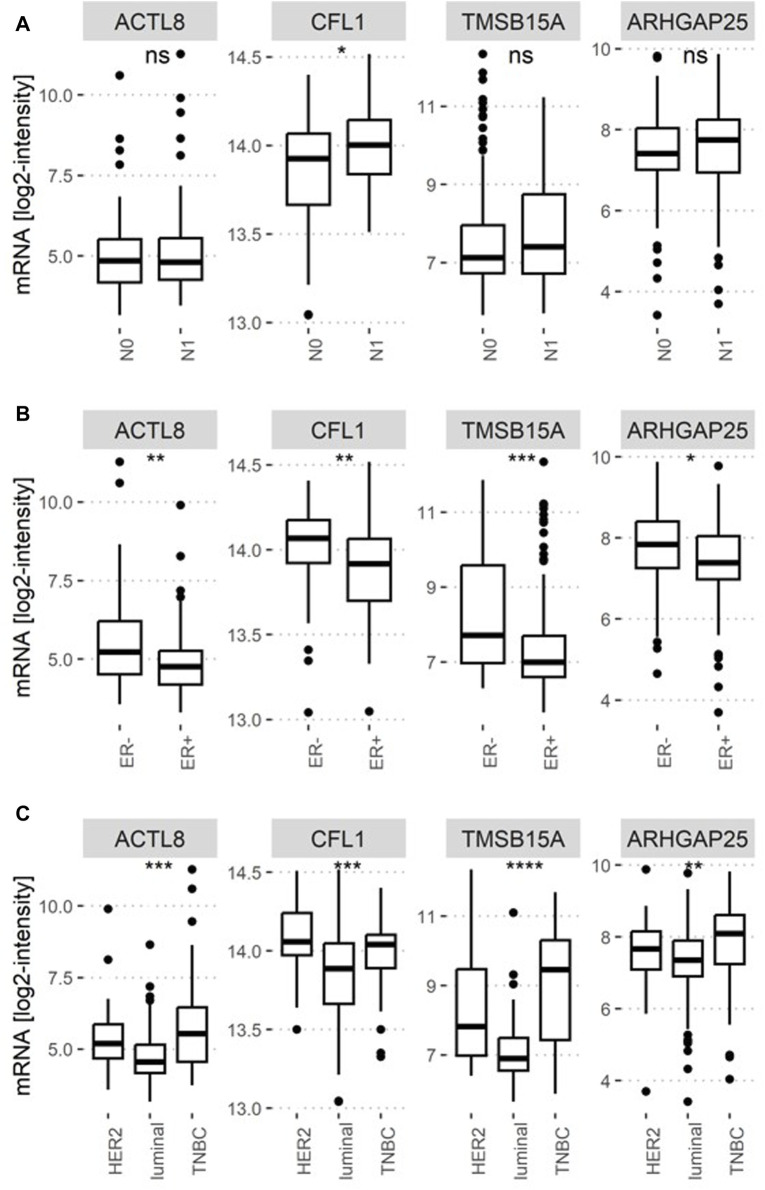
Distributions of ABPs encoding gene expression by lymph node and estrogen receptor status at baseline and molecular breast cancer subtype. (A) The mRNA levels of ABPs were tested for differences regarding the ability of cancer cells to form lymph node metastasis; N0: No lymph node involvement; N1: At least one lymph node is involved. **(B)** Differential expression between estrogen receptor (ER) expression positive and negative breast cancer samples. **(C)** Differences between Her2-positive, luminal, and triple-negative breast cancer subtypes. In A-B, differences in the mean mRNA expression were tested using Wilcoxon tests, and in C, Kruskal–Wallis tests were performed. Statistical significance is indicated by ns (not significant) *p*-value >0.05, * *p*-value ≤0.05, ** *p*-value ≤0.01, *** *p*-value ≤0.001, **** *p*-value ≤0.0001.

In order to analyze whether the genes encoding ABPs are preferentially expressed by certain cancer subtypes, differential mRNA expression analyses were performed for lymph node status (N0: no lymph nodes are affected; N1: at least one lymph node is affected), estrogen receptor status (ER+/−: estrogen receptor-positive/negative) and the molecular subtype at baseline (luminal, HER2-positive, triple-negative breast cancer). As shown in Figure 4, all ABPs significantly correlating with the ability of the primary tumor to form distant metastases were preferentially expressed by HER2-positive and triple-negative breast cancer, even *ARHGAP25* showing an inverse correlation with metastasis-free survival probability. Also, the mRNA levels of *CFL1, TMSB15A, ACTL8,* and *ARHGAP25* were higher in ER^−^ than ER^+^. However, only CFL1 mRNA was significantly higher in pN1 than in pN0 tumors.

In conclusion, the ABPs whose mRNA level is associated with a high metastatic potential are preferentially expressed by more aggressive breast cancer subtypes.

### Validation of the identified genes in other breast cancer cohorts

In order to validate the candidate genes in other breast cancer cohorts, their expression was tested for associations with metastasis-free survival probability in the GEO data sets GSE11121 (Schmidt et al., 2008), GSE6532 (Loi et al., 2007), GSE 2034 (Wang et al., 2005), and GSE21653 (Sabatier et al., 2011). Here, GSE6532 and GSE1653 included the same patient information as our “discovery” cohort, while only lymph node-negative patients were included in GSE11121 and GSE20234. Furthermore, GSE2034 only contained samples from ER-positive tumors, while in GSE11121, no data about the hormone status was available (Table 1). One of four candidate genes was captured in GSE6532, *CFL1*.

**TABLE 1:** Clinical data of Discovery and the publicly available breast cancer cohorts GSE11121, GSE6532, GSE2034, GSE21653.

Validation studies					
Study	Discovery	GSE11121	GSE6532	GSE2034	GSE21653
N	181	200	293	286	252
N relapse	76 (42.0)	46 (23.0)	68 (23.2)	107 (37.4)	83 (32.9)
Metastasis free survival [months], mean (sd)	128.1 (78.6)	93.9 (50.7)	78.0 (43.4)	77.5 (42.3)	60.0 (41.4)
Age, mean (sd)	55.5 (11.1)	-	59.2 (11.6)	-	55.3 (13.6)
Lymph node positive, n (%)	56 (30.9)	0 (0.0)	77 (26.3)	0 (0.0)	133 (52.8)
ER positive, n (%)	137 (75.7)	156 (78)	248 (84.6)	209 (73.1)	140 (56.0)
PR positive, n (%)	116 (64.1)	130 (65)	118 (91.5)	165 (58)	126 (50.4)
Grade, n (%)					
I	20 (11.0)	29 (14.5)	62 (21.1)	-	43 (17.3)
II	80 (44.2)	136 (68.0)	140 (47.8)	-	84 (33.9)
III	92 (50.8)	35 (17.5)	56 (19.1)	-	121 (48.8)
Therapy	Radiotherapy	no systemic (radiotherapy)	Majority tamoxifen	no systemic (radiotherapy)	Radiotherapy
	Hormontherapy				Hormontherapy
	Chemotherapy				Chemotherapy

**FIGURE 5:**
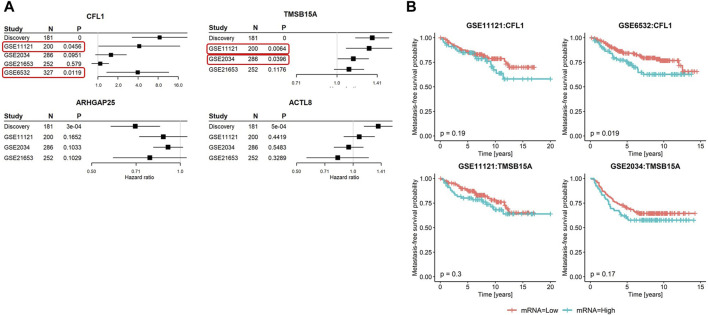
Summary of metastasis-free survival analyses in the discovery and validation cohorts. (A) Forest plots of the four candidate genes showing hazard ratios, 95% confidence intervals, *p*-values, and sample sizes of the discovery and three to four published breast cancer cohorts. In GSE6532, only the mRNA of CFL1 was captured. **(B)** Kaplan–Meier curves stratified based on the second tertile of candidate genes meeting the validation criteria in relation to metastasis-free survival probabilities. Red rectangles highlight the validation cohorts that led to the successful validation of the corresponding genes.

Among the four initially identified ABPs in the discovery cohort (Figure 1), two met the replication criteria with a *p*-value ≤0.05 and a consistent direction of the hazard ratio compared to the discovery in at least two further cohorts (Figure 5A). The estimated hazard ratio of *CFL1* mRNA was 8.4 (95% CI: 3.2–21.8) in the discovery, 4.2 (95% CI: 1.0–16.9) in GSE11121, and 4.0 (95% CI: 1.4–11.5) in GSE6532, while that of *TMSB15A* was 1.4 (95% CI: 1.2–1.6) in the discovery, 1.3 (95% CI: 1.1–1.6) in GSE11121, and 1.1 (95% CI: 1.0–1.3) in GSE 2034. On the other hand, Kaplan–Meier analysis revealed that only CFL1 showed a significant correlation with low metastasis-free survival probability in GSE6532 (Figure 5B).

**FIGURE 6:**
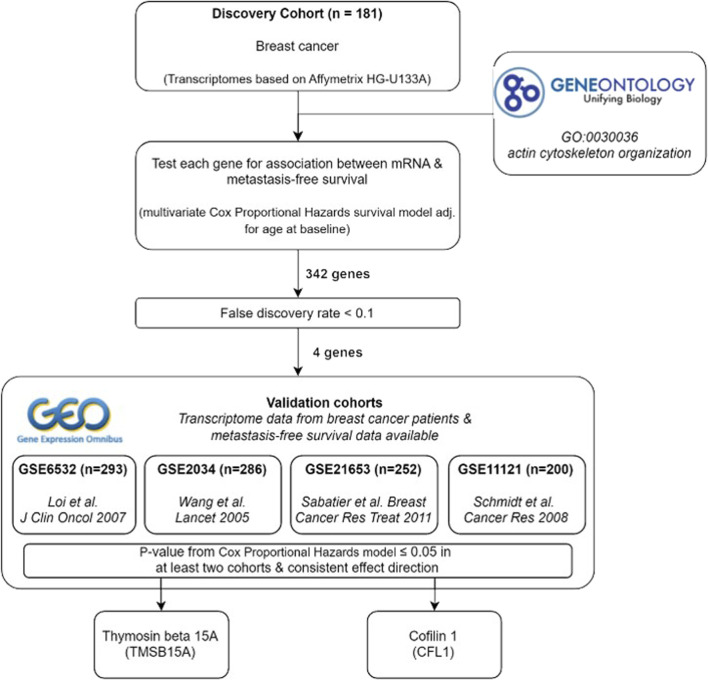
Summary of study design and results.

In summary, among all ABPs analyzed in this study, a high mRNA level of Cofilin 1 showed the strongest association with the metastatic potential of breast cancer tumors (Figure 6).

## Discussion

It has already been shown that a high level of several actin-binding proteins (ABP) correlates with breast cancer metastases (Izdebska et al., 2020). However, until now, a ranking of these proteins based on their importance in association with cancer malignancy has not been established. Because this approach enables the identification of the best-suited targets for cancer therapy, an unbiased bioinformatic method using five independent transcriptome data sets was conducted in this study to identify those ABPs with key roles in highly metastatic breast cancer cells. For this purpose, we selected all genes from the Gene Ontology term actin cytoskeleton organization and manually curated a list of ABPs directly interacting with actin (ABPs) or belonging to the RhoGTPases and their regulators. To analyze potential associations with the metastatic potential of breast cancer cells, their mRNA levels were correlated with the metastasis-free survival probability of breast cancer patients from our in-house cohort. Thereby, we identified four different genes, among which three were significantly associated with shorter (*CFL1, TMSB15A,* and *ACTL8*) and one with longer metastasis-free survival (*ARHGAP25*). In subsequent Kaplan–Meier analysis, two genes remained significant: *CFL1 and TMSB15A.*

Rho GTPase activating protein 25 (ARHGAP25) was the only ABP associated with a low metastatic potential of breast cancer cells. ARHGAP25 is a negative regulator of the RhoGTPase Rac1, and in neutrophils, it negatively regulates phagocytosis by controlling the actin cytoskeleton (Csepanyi-Komi et al., 2012). In lung cancer cells, a high ARHGAP25 level exhibits tumor suppressor activity and thus increases the overall survival of lung cancer patients (Xu et al., 2019; Shi et al., 2022). Also, in breast cancer tissues, ARHGAP25 is downregulated relative to normal tissues, and its knockdown in breast cancer cell lines decreased malignancy (Han et al., 2023). These data indicate that ARHGAP25 may exhibit tumor suppressor activity. However, our analysis did not show a significant association in Kaplan–Meier analysis, and the aggressive breast cancer phenotypes (Her2+ and triple-negative) showed a slightly increased ARHGAP25 mRNA level compared to luminal breast cancer. Furthermore, the result obtained from our discovery cohort could not be validated in further breast cancer cohorts. Therefore, future studies are necessary to validate the prognostic value of ARHGAP25 in breast cancer.

*CFL1, TMSB15A*, and *ACTL8* were significantly associated with a high metastatic potential. Among these, *CFL* exhibited the highest hazard ratio of 8, and only *CFL1* mRNA was significantly increased in pN1 compared to pN0 tumors. Cofilin 1 (CFL1) is an F-actin severing protein that promotes actin turnover and is, therefore, essential for cell motility. According to the current model, the actin subunits provided by Cofilin 1-mediated actin cleavage are used by the Arp2/3 complex and by VASP to elongate F-actin at the tips of cellular protrusions and thereby drive migration and invasion of cancer cells (Wang et al., 2007). Moreover, many studies showed a clear correlation between high CFL1 expression and aggressive progression of cancer cells [reviewed in Wang et al. (2007), Coumans et al. (2018), Sidani et al. (2007), Xu et al. (2021)], supporting our finding that CFL1 highly significantly correlates with the ability of breast cancer cells to form metastases.

In addition to *CFL1*, the mRNAs of *TMSB15A* and *ACTL8* show significant HR results.

Like *CFL1*, they were more highly expressed in subtypes with more aggressive behavior, but their mRNA level was not significantly different between pN0 and pN1 tumors. Thymosins are G-actin sequestering proteins, saving a free cellular G-actin pool and thereby inhibiting F-actin polymerization. Together with the G-actin-binding protein Profilin, which promotes actin polymerization, the Thymosins control directed F-actin elongation, and that is why their binding to G-actin is tightly regulated by cellular stimulation. Darb-Esfahani et al. (2012) revealed that TMSB15A is a predictor of chemotherapy response in triple-negative breast cancer, and Zhang et al. (2017) found elevated levels of TMSB10 in breast cancer tissues as well as a significant correlation with metastasis status and poor prognosis. In melanoma cells, TMSB4 regulates focal adhesion formation as well as migration and invasion (Makowiecka et al., 2019). Thus, different Thymosin isoforms seem to be involved in the malignant progression of breast cancer cells. The cancer Testis antigen ACTL8 shows a similar hazard ratio as TMSB15A (1.36) and is co-expressed with CFL1 and TMSB15A, but it was not found to be significant in Kaplan–Meier analysis. ACTL8 is upregulated in different tumor types (Yang et al., 2022), and its knock-down inhibited malignancy of lung cancer A549 cells (Ma et al., 2019) as well as of triple-negative breast cancer cells (Fan et al., 2021). However, to our knowledge, its role in actin dynamics has not been investigated. Furthermore, among the ABPs whose high mRNA level showed an association with a high metastatic potential of the primary tumors, ACTL8 exhibited the lowest significance. Thus, similar to ARHGAP25, its prognostic value for breast cancer patients is rather weak.

Among our four initially identified hits, only TMSB15A and CFL1 showed a significant association with metastasis-free survival in further validation cohorts. Both genes could be validated in the cohort GSE11121, which includes only nodal-negative patients who did not receive any systemic therapy. Interestingly, TMSB15A also showed a significant prognostic value in a second cohort (GSE 2034) with similar characteristics, and for CFL1, we observed a strong trend with a borderline significant value in this cohort. GSE2034 includes mainly ER-positive, luminal breast cancer patients. Additionally, a significant association with increased metastatic potential, identified in Kaplan–Meier and log-rank tests, could be determined for CFL1 in the GSE6532 cohort, showing similar characteristics to the discovery cohort. This cohort includes both luminal and basal subtype patients treated with standard hormonal therapy and chemotherapy.

## Conclusion

In conclusion, among the ABPs identified in this study as being associated with a strong metastatic potential of breast cancer cells, CFL1 showed the most robust results. It exhibited the highest hazard ratio in our in-house and two published breast cancer cohorts. Also, a high *CFL1* mRNA level was associated with malignant breast cancer subtypes, and *CFL1* expression was highest in breast cancer populations that formed metastases within 5 years. In the literature, CFL1 has been described to play a key role in the malignant progression of cancer cells (reviewed in Wang et al. (2007), Coumans et al. (2018), and Xu et al. (2021)). Thus, among the ABPs associated with the malignant progression of cancer cells, Cofilin 1 seems to be a key player, and it would be of high interest to develop therapeutics specifically targeting Cofilin 1.

## Data Availability

The datasets presented in this study can be found in online repositories. The names of the repository/repositories and accession number(s) can be found in the article/Supplementary Material.
